# Prognostic Significance of Interim PET/CT in the First-Line Treatment of Follicular Lymphoma Patients, a Single-Center Experience

**DOI:** 10.3390/cancers17010073

**Published:** 2024-12-29

**Authors:** Evelin Kiss, Zsófia Simon, Árpád Illés, Ádám Jóna

**Affiliations:** 1 Department of Hematology, Faculty of Medicine, University of Debrecen, 4032 Debrecen, Hungary; evlnkiss92@gmail.com (E.K.); zsocogo@gmail.com (Z.S.); illesarpaddr@gmail.com (Á.I.); 2 Doctoral School of Clinical Medicine, University of Debrecen, 4032 Debrecen, Hungary

**Keywords:** follicular lymphoma, lymphoma, interim, PET/CT, prognostic factor, Deauville score, SUVmax

## Abstract

**Introduction**: Follicular lymphoma (FL) is a heterogeneous disease, and identifying high-risk patients early is crucial for optimal management. This study aimed to evaluate the prognostic significance of interim positron emission tomography/computed tomography (PET/CT) in newly diagnosed FL patients undergoing first-line treatment. **Methods**: This single-center, retrospective study included 103 patients with newly diagnosed FL who underwent interim PET/CT. The primary endpoint was progression-free survival (PFS). The prognostic value of positive interim PET/CT, Deauville scores, and maximum standardized uptake values (SUVmax) were analyzed among other clinical features. **Results**: A total of 30 patients (29.1%) were deemed interim PET/CT-positive. The interim PET/CT-positive group demonstrated a significantly shorter median PFS compared to the interim PET/CT-negative group (17 months vs. not reached, respectively; *p* < 0.0001). Similarly, patients with Deauville scores of 1–3 had better PFS, with median survival not yet reached, while those with higher Deauville scores exhibited poorer progression-free survival and a median survival of 18 months. Notably, patients with an interim PET/CT SUVmax exceeding 3.365 experienced significantly shorter median survival compared to those with lower values. **Discussion**: Our findings support the use of interim PET/CT as a prognostic tool in FL. These results are consistent with other studies, although some debate exists regarding the optimal PET/CT metric. Further research is needed to validate these findings and explore personalized treatment strategies based on interim PET/CT results.

## Simple Summary

Our research looks at a special type of scan, called a PET/CT scan, taken midway through treatment for a blood cancer called follicular lymphoma. Doctors want to know whether this mid-treatment scan can predict how well patients will do in the long run. The study found that patients with a “positive” mid-treatment scan, meaning their cancer is still active, tend to have worse outcomes. This is important because it could help doctors identify high-risk patients early on and potentially adjust their treatment. While other studies have looked at this, this research adds to the evidence and suggests that this mid-treatment scan could be a valuable tool for managing follicular lymphoma. However, more research is needed to confirm these findings and figure out the best way to use this information to improve patient care.

## 1. Introduction

Follicular lymphoma (FL) is a type of non-Hodgkin’s lymphoma characterized by the proliferation of follicular center B cells [1]. It is the second most common subtype of non-Hodgkin’s lymphoma [2], and its management and prognosis have been the subject of extensive research. Follicular lymphoma exhibits substantial biological and clinical heterogeneity, leading to diverse outcomes among individual patients. The incorporation of the anti-CD20 monoclonal antibody rituximab has significantly improved the results of FL treatment [3], with the median overall survival now approaching 20 years. Nevertheless, the majority of patients ultimately experience disease relapse. Ongoing research focuses on the ability to deliver personalized treatment regimens based on individualized risk assessment of patients.

The Follicular Lymphoma International Prognostic Index (FLIPI) [4] and the Follicular Lymphoma International Prognostic Index 2 (FLIPI2) [5] are two well-established prognostic models that have been widely used to stratify patients with follicular lymphoma based on their risk of disease progression and overall survival. These models incorporate factors such as age, disease stage, serum lactate dehydrogenase level, hemoglobin level, and the number of involved nodal sites to estimate a patient’s prognosis and guide treatment decisions. The FLIPI and FLIPI2 have demonstrated their utility in predicting outcomes in FL, and they continue to be important tools in the management of this disease. Others, like PRIMA PI (Prognostic Index) [6] and m7 FLIPI [7], include additional biomarkers and genetic factors that may provide more personalized prognostic information to further refine risk stratification, such as the proliferation marker Ki-67 (marker of proliferation Kiel 67), the anti-apoptotic protein BCL2 (B-cell leukemia/lymphoma 2), and various molecular genetic abnormalities [8,9].

However, despite their widespread use, these prognostic indices present limitations in fully capturing the clinical heterogeneity observed in follicular lymphoma, as some patients may experience indolent disease courses for decades without requiring treatment; others may have more aggressive disease that is refractory to standard therapies.

One important aspect of FL management is the use of positron emission tomography and computed tomography imaging, particularly in the assessment of treatment response and prediction of prognosis. The use of interim positron emission tomography and computed tomography (PET/CT) imaging during the course of first-line treatment has emerged as a potential tool to further refine prognostic assessment and guide management decisions FL [10]. However, the prognostic significance of interim PET/CT in the first-line treatment of follicular lymphoma patients remains an active area of investigation.

This study aims to investigate our patient data in the context of the current evidence on the prognostic value of interim PET/CT in the management of follicular lymphoma patients receiving first-line treatment.

## 2. Methods

A retrospective analysis of patients with newly diagnosed FL (but grade 3b cases were excluded) who underwent first-line treatment at the University of Debrecen Department of Hematology between May 2009 and March 2024 was conducted. Biopsy specimens were obtained from the most readily accessible site, as histological confirmation is required prior to ordering PET/CT imaging.

FL is graded based on the number of centroblasts observed in a high-power microscopic field. Grade 1 FL is characterized by fewer than 5 centroblasts per high-power field, grade 2 has 5–15 centroblasts per high-power field, grade 3A has more than 15 centroblasts per high-power field, and grade 3B is defined by the presence of sheets of centroblasts [11].

Patients were included if they underwent an interim PET/CT scan performed after three to four cycles of therapy. Data on patient demographics (age and sex), disease characteristics (histology, stage, presence or absence of B symptoms, absolute lymphocyte (Ly) and monocyte (Mo) counts, and lymphocyte/monocyte (Ly/Mo) ratio), FLIPI scores, treatment regimens, PET/CT findings (Deauville score and maximum standardized uptake value (SUVmax)), changes in interim PET/CT SUVmax from the baseline, and clinical outcomes were collected. The prognostic significance of interim PET/CT findings, defined as the PET/CT performed during first-line treatment, was analyzed in relation to progression-free survival (PFS). No treatment change was performed according to the results of interim PET/CT, which were obtained as an institutional endeavor to document more precisely the course of treatment.

All patients provided written informed consent before treatment initiation to allow for the retrospective collection and publication of their data, in accordance with the Declaration of Helsinki. This retrospective analysis was approved by the Regional and Institutional Research Ethics Committee of the University of Debrecen (DE RKEB/IKEB 5694–2021). The patients were treated based on current institutional guidelines. Treatment was initiated for patients who met the Groupe d’Etude des Lymphomes Folliculaires criteria [12]. Grade 1 and 2 patients were given R-CVP (rituximab, cyclophosphamide, vincristine, and prednisolone) until 2015, after which bendamustine became widely available. Grade 3a patients received R-CHOP (cyclophosphamide, doxorubicin, vincristine, and prednisolone) chemotherapy. Additionally, FLIPI high-risk patients have been administered obinutuzumab since 2018 or as part of clinical trials.

PET/CT has been a standard imaging modality employed at the University of Debrecen since May 2009. However, the utilization of interim PET/CT scans during treatment was not a routine practice. The interim PET/CT scans were assessed using the Deauville five-point scale, which is widely accepted for evaluating response to therapy in lymphoma. The Deauville five-point scale is used to interpret PET scans in lymphoma by comparing FDG uptake in lesions to the liver and mediastinum. A Deauville score (DS) 1 indicates no uptake or uptake that is less intense than the mediastinum, while a DS 2 signifies uptake equal to or slightly greater than the mediastinum but less than the liver. A DS 3 represents uptake slightly greater than the liver. A DS 4 shows uptake moderately higher than the liver, and a DS 5 indicates uptake markedly higher than the liver or new lesions. If there is focal uptake in the liver, the spleen is used as the reference organ instead. Visual interpretation is used for the DS, and interobserver variability can occur, so experienced nuclear medicine physicians should perform the assessment [13]. Deauville scores of 1–3 were considered a negative examination, while scores of 4–5 were considered a positive response [14]. Before the introduction of Deauville scores, a comparison was performed of the involved lymph node and the mediastinal activity. Lower activity than the mediastinal activity was considered negative, while a higher activity than the mediastinal activity was considered positive. PFS was defined as the time from the start of first-line treatment to the first occurrence of disease progression, relapse, or death from any cause.

The PET/CT examinations followed a detailed institutional protocol. Patients were required to fast for 6 h prior to the procedure, and their blood glucose level was checked upon arrival, with the test only performed if the level was below 12.5 mmol/L. Patient weight and height were also recorded. Radiopharmaceuticals were administered using a specific injector, Intego, with the dose based on the patient’s body weight (4.4 MBq/kg). The uptake time was 60 min, during which patients were required to rest, avoiding activities such as watching television or listening to intensive music. Patients were instructed to drink 1 L of water before and during the waiting time and to empty their bladders prior to the scan. The PET/CT acquisition process involved a CT localizer, whole-body CT, and PET imaging, with specific parameters for both modalities. The PET/CT scans were interpreted by experienced nuclear medicine specialists who were blinded to the clinical data.

The maximum standardized uptake value was measured using the Interview Fusion software version 3.03.077.0007, which performs cross-validation with the Philips ISP system. The SUVmax was calculated based on the patient’s body weight, and the SUV units were g/mL. A nuclear medicine specialist identified and measured the lesions on the fused PET/CT images, placing regions of interest on the areas with the most intense radiotracer uptake.

A univariate analysis was conducted to evaluate factors potentially impacting survival. Continuous variables were transformed into discrete variables, either by exceeding standard values or by determining data cut-off points using receiver operating characteristic curves. The state variables were defined as the events associated with PFS. A multivariable Cox regression model with the Enter method was utilized to calculate the hazard ratio. Survival was assessed via the Kaplan–Meier approach, and the log-rank test was used to compare survival curves. Statistical significance was set to *p* < 0.05.

## 3. Results

We examined patients with newly diagnosed FL who underwent first-line treatment at our institution between May 2009 and March 2024 and had an available interim PET/CT scan. The median follow-up time was 40 months, ranging from 2 to 199 months. Table 1 presents the characteristics of the 103 FL patients included in the study. The median age at diagnosis was 54 years, with a range of 25 to 83 years. There were slightly more females than males in the study. The majority of patients had Grade 2 or 3a follicular lymphoma. Most patients presented with advanced-stage disease, with 36 patients in Stage 3 and 55 in Stage 4. Thirty-three patients presented with B symptoms. The FLIPI risk distribution was relatively even, with 21 low, 34 intermediate, and 44 high-risk patients. The majority of patients received R-CHOP, followed by anti-CD20 (most of them rituximab, while one patient received obinutuzumab as part of a clinical trial) bendamustine treatment.

A univariate analysis was performed (Table 2), examining the association between various factors and PFS in FL patients. Patients with positive interim PET/CT scans obtained a significantly worse PFS compared to those with negative scans (*p* < 0.0001). Higher Deauville scores and interim PET/CT SUVmax values were also significantly associated with worse PFS (*p* < 0.0001 for both). Other factors like age, sex, histology grade, B symptoms, FLIPI score (low, intermediate, or high), lymphocyte or monocyte count, lymphocyte/monocyte ratio, changes in SUVmax values from the baseline, and treatment type did not show statistically significant associations with PFS in this univariate analysis.

**Figure 1:**
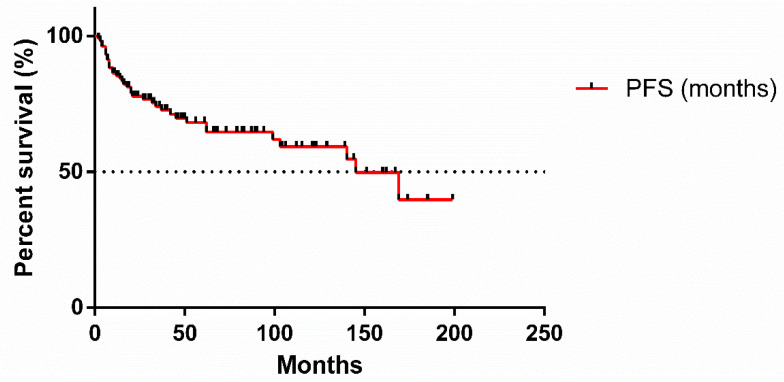
Progression-free survival of follicular lymphoma patients.

Figure 1 illustrates the PFS of FL patients involved in this analysis. The median PFS was 145 months, indicating that half of the patients remained progression-free for over 12 years. The 5-year PFS was 68.12%, while the 10-year PFS was 59.24%.

**Figure 2:**
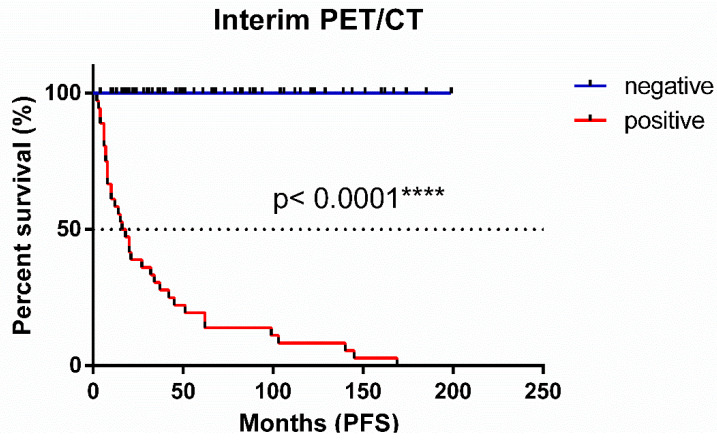
Progression-free survival of interim PET/CT-positive and -negative follicular lymphoma patients.

Kaplan–Meier curves for the progression-free survival of patients stratified by interim PET/CT results are shown at Figure 2. The curves demonstrate a significant difference in PFS based on interim PET/CT findings (*p* < 0.0001). Patients with negative interim PET/CT scans showed superior progression-free survival, with the median survival not yet reached. In contrast, patients with positive interim PET/CT had a significantly shorter median PFS of 17 months.

**Figure 3:**
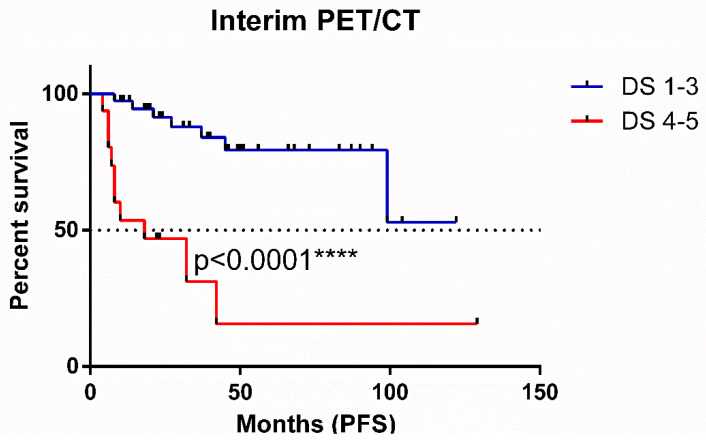
Progression-free survival of follicular lymphoma patients according to Deauville scores.

Interim PET/CT Deauville scores demonstrated a significant impact on PFS, as illustrated by the Kaplan–Meier curves in Figure 3 (*p* < 0.0001). Patients achieving Deauville scores of 1–3 experienced superior PFS, with median survival not yet reached. In contrast, those with higher scores (4–5) had inferior PFS and a median survival of 18 months.

**Figure 4:**
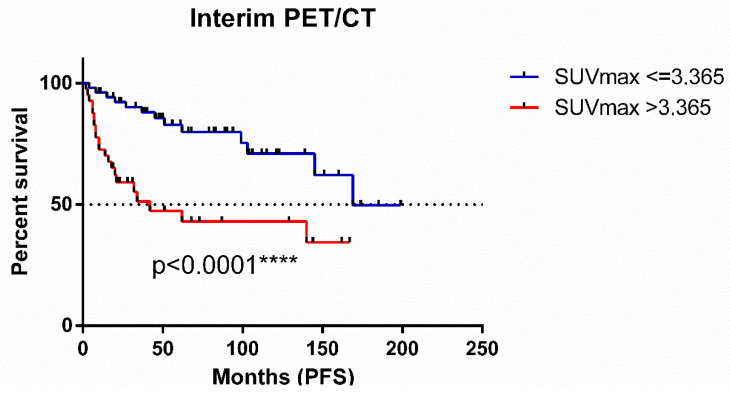
Progression-free survival of follicular lymphoma patients with a cut-off of 3.365 SUVmax value based on receiver operating characteristic analysis.

We performed an ROC (receiver operating characteristic) analysis (Figure 4), which revealed a powerful prognostic indicator for PFS based on interim PET/CT SUVmax results. With a significant difference observed at a cut-off of 3.365 (*p* < 0.0001), the figure demonstrates a stark contrast in outcomes. Patients exhibiting an SUVmax of 3.365 or lower experienced markedly superior PFS, reflected in a median survival of 169 months. Conversely, those with SUVmax exceeding 3.365 faced a significantly shorter median survival of 42 months. This finding underscores the potential of interim PET/CT SUVmax, particularly at this identified threshold, to effectively stratify patients and predict long-term outcomes.

## 4. Discussion

The results of this single-center, retrospective study highlight the prognostic significance of interim PET/CT in patients with newly diagnosed follicular lymphoma undergoing first-line treatment. We found that positive interim PET/CT scans, higher Deauville scores, and elevated SUVmax values were all strongly associated with inferior progression-free survival. Our findings are consistent with the growing body of evidence supporting the prognostic value of interim PET/CT in the management of follicular lymphoma. Given the heterogeneous nature of follicular lymphoma, accurately identifying high-risk patients early in the disease course is crucial.

PET/CT plays an essential role in managing FL. While restaging PET/CT is a strong predictor of outcomes, the role of interim PET/CT in guiding therapy requires further investigation. More research is needed to personalize treatment based on PET/CT findings and optimize outcomes for FL patients; furthermore, there is no consensus on how to manage patients who are interim PET/CT-positive. Trials are ongoing to determine whether changing therapy based on interim PET improves outcomes [15].

A recent meta-analysis identified limitations in the existing evidence regarding the use of interim PET/CT in FL. The included studies generally exhibited poor methodological rigor, with most being retrospective in nature and utilizing small sample sizes, as well as heterogeneous methods. Furthermore, many of these studies employed the International Harmonisation Project criteria for interpreting interim PET/CT scans, rather than the Deauville scoring system, which is the current standard of care in clinical practice [16,17].

A recent ASH (American Society of Hematology) abstract from 2022 [18] reported findings analogous to those of the present study, highlighting the prognostic value of interim PET/CT in FL. Both investigations centered on PFS as the primary endpoint and determined that interim PET/CT results significantly correlated with PFS. Additionally, both studies employed Deauville scoring and observed that higher scores were associated with inferior PFS. However, the prior work involved 143 patients and documented a median follow-up duration of 67 months, further validating interim PET/CT Deauville score of 4–5 as an independent predictor of outcomes.

Merryman et al. [19] conducted a study on a cohort of FL patients treated at two institutions, focusing on the prognostic value of interim PET/CT scans performed after three cycles of therapy. Their findings align with the results of the present study. The researchers utilized both the Deauville score and the change in SUVmax from the baseline to interim PET/CT to assess treatment response. Their analysis revealed that interim PET/CT scan results were significantly associated with PFS. Patients with negative interim PET/CT scans exhibited significantly better PFS compared to those with positive scans. Furthermore, a 75% reduction in SUVmax from the baseline to interim PET/CT was also identified as a strong predictor of PFS, with patients who achieved this threshold demonstrating significantly better outcomes. The study suggests that interim PET/CT scans may provide similar prognostic information to end-of-treatment PET/CT scans in the management of follicular lymphoma.

Another recent study [20] investigating interim PET/CT in follicular lymphoma patients used the Deauville score to assess response. Contrary to our findings and the ASH abstract [18], this study found no significant association between Deauville scores and PFS. However, the change in SUVmax from the baseline to interim PET/CT was significantly associated with PFS. Patients with a larger decrease in SUVmax achieved better outcomes. While both studies investigated interim PET/CT in FL, the conflicting findings regarding the Deauville score highlight the complexity of using interim PET/CT for prognostication. They suggest that ΔSUVmax (the change in SUVmax from the baseline to interim PET/CT) might be a more reliable predictor than the Deauville score alone, warranting further investigation. The difference is also that they focused solely on patients treated with R-CHOP, while our study included various treatments.

A letter to the editor [21] raised concerns about the study detailed in the previous paragraph [20]. The author disagrees with the study’s conclusion that interim PET/CT lacks prognostic value for predicting PFS in FL. The author points out several limitations of the original study, including that only 30 patients were included, which might not be representative. The timing of interim PET/CT scans varied, potentially affecting the results. The author shares their own findings based on a larger patient population with interim PET/CT performed strictly after the second therapy cycle. Their results showed significant differences in both PFS and overall survival based on Deauville scores.

## 5. Conclusions

The data from this study indicate that patients exhibiting positive interim PET/CT findings, higher Deauville scores, and elevated interim PET/CT SUVmax values experienced inferior PFS outcomes. This finding is consistent with other studies despite some conflicting evidence regarding the predictive value of the Deauville score. Nevertheless, the change in SUVmax values may also be an important prognostic factor. Additional research is required to better elucidate the prognostic significance of interim PET/CT in FL, including the potential for tailoring therapy based on these findings.

## 6. Limitations

This study constituted a retrospective, single-institution analysis with patients treated over a long period of time. Furthermore, the study population received varied first-line treatments. Variation in the utilization of rituximab maintenance therapy, reflecting evolving practice patterns during the study period, represents a potential confounding factor. A remarkable limitation is the histological grading of the tumors, with 10.7% of cases having an undefined grade. The differences observed in the PFS curves across Figure 2, Figure 3 and Figure 4 can be attributed to the availability of data for each patient. Additionally, the Deauville scoring system was introduced in 2014 [22], meaning that, for earlier patients, only SUVmax values were available for analysis.

## Figures and Tables

**Table 1:** Patient characteristics.

Patient number		103
Male/female		45/58
Age (yrs, median, range)		54 (25–83)
Grade	1	30
	2	36
	3a	26
	ND	11
Stage	2	12
	3	36
	4	55
B-symptoms		33
FLIPI risk	low	21
	intermediate	34
	high	44
	unknown	4
Treatment	antiCD20-bendamustin	37
	R-CVP	9
	R-CHOP	57
	rituximab maintenance	65

ND—not defined.

**Table 2:** Univariate analysis of factors and progression-free survival in follicular lymphoma patients.

			95% CI for HR		
		HR	Lower	Upper	Sig.
Age		0.99	0.96	1.02	0.501
Sex		0.98	0.501	1.918	0.954
Histology	Grade 1	0.392	0.131	1.173	0.094
	Grade 2	0.803	0.31	2.077	0.651
	Grade 3a	0.561	0.194	1.618	0.285
	ND				0.296
Stage	2	7.126	0.816	62.222	0.076
	3	1.665	0.211	13.177	0.628
	4	2.419	0.323	18.115	0.39
B-symptoms		0.945	0.459	1.946	0.878
FLIPI		1.333	0.163	10.907	0.789
Treatment	R-bendamustin				0.932
	R-CHOP	1.156	0.538	2.484	0.71
	R-CVP	1.076	0.329	3.523	0.904
R maintenance		0.528	0.226	0.1232	0.14
#Ly		0.629	0.18	2.201	0.468
#Mo		0.943	0.017	51.483	0.977
Ly/Mo		0.774	0.371	1.614	0.495
**Interim PET pos**		**4.815**	**2.398**	**9.669**	**0.0001**
**Deauville score**		**3.521**	**1.928**	**6.431**	**0.0001**
**Interim PET SUVmax**		**1.15**	**1.084**	**1.221**	**0.0001**
Delta SUVmax		1.436	0.678	3.043	0.345

#Ly—absolute lymphocyte count; #Mo—absolute monocyte count.

## Data Availability

Data us available as supplementary material (Supplemantary File S1).

## Supplementary Materials

The following supporting information can be downloaded at: https://www.mdpi.com/article/10.3390/cancers17010073/s1. Supplemantary File S1: original data.
